# GammaTile® brachytherapy in the treatment of recurrent glioblastomas

**DOI:** 10.1093/noajnl/vdab185

**Published:** 2021-12-27

**Authors:** Dominic J Gessler, Elizabeth C Neil, Rena Shah, Joseph Levine, James Shanks, Christopher Wilke, Margaret Reynolds, Shunqing Zhang, Can Özütemiz, Mehmet Gencturk, Mark Folkertsma, W Robert Bell, Liam Chen, Clara Ferreira, Kathryn Dusenbery, Clark C Chen

**Affiliations:** 1 Department of Neurosurgery, University of Minnesota, Minneapolis, Minnesota, USA; 2 Department of Neurology, University of Minnesota, Minneapolis, Minnesota, USA; 3 Department of Oncology, North Memorial Health, Robbinsdale, Minnesota, USA; 4 Department of Oncology, Fairview Cancer Care, Minneapolis, Minnesota, USA; 5 Department of Radiation Oncology, University of Minnesota, Minneapolis, Minnesota, USA; 6 Department of Radiology, University of Minnesota, Minneapolis, Minnesota, USA; 7 Department of Pathology, University of Minnesota, Minneapolis, Minnesota, USA

**Keywords:** brachytherapy, GammaTile®, O^6^-Methylguanine-DNA methyltransferase (MGMT), recurrent glioblastoma

## Abstract

**Background:**

GammaTile® (GT) is a recent U.S. Food and Drug Administration (FDA) cleared brachytherapy platform. Here, we report clinical outcomes for recurrent glioblastoma patients after GT treatment following maximal safe resection.

**Methods:**

We prospectively followed twenty-two consecutive Isocitrate Dehydrogenase (IDH) wild-type glioblastoma patients (6 O^6^-Methylguanine-DNA methyltransferase methylated (MGMTm); sixteen MGMT unmethylated (MGMTu)) who underwent maximal safe resection of recurrent tumor followed by GT placement.

**Results:**

The cohort consisted of 14 second and eight third recurrences. In terms of procedural safety, there was one 30-day re-admission (4.5%) for an incisional cerebrospinal fluid leak, which resolved with lumbar drainage. No other wound complications were observed. Six patients (27.2%) declined in Karnofsky Performance Score (KPS) after surgery due to worsening existing deficits. One patient suffered a new-onset seizure postsurgery (4.5%). There was one (4.5%) 30-day mortality from intracranial hemorrhage secondary to heparinization for an ischemic limb. The mean follow-up was 733 days (range 279–1775) from the time of initial diagnosis. Six-month local control (LC_6_) and twelve-month local control (LC_12_) were 86 and 81%, respectively. Median progression-free survival (PFS) was comparable for MGMTu and MGMTm patients (~8.0 months). Median overall survival (OS) was 20.0 months for the MGMTu patients and 37.4 months for MGMTm patients. These outcomes compared favorably to data in the published literature and an independent glioblastoma cohort of comparable patients without GT treatment.

**Conclusions:**

This clinical experience supports GT brachytherapy as a treatment option in a multi-modality treatment strategy for recurrent glioblastomas.

**Key Points**

- We report the first clinical series of GT-treated recurrent glioblastoma patients.
- The safety profile of GT implant is comparable to those who underwent re-resection.
- Local control and survival outcome are favorable, especially in MGMTm patients.

**Importance of the Study**

Gammatile® (GT) is a brachytherapy platform consisting of Cesium-131 (^131^Cs) seeds embedded with an absorbable collagen matrix that received U.S. FDA clearance to treat recurrent brain tumors in late 2018. Here, we report the first clinical series describing the use of GT since its approval in recurrent glioblastoma patients. With a mean follow-up of 733 days from initial diagnosis, we prospectively followed 22 IDH wild-type glioblastomas treated with maximal safe resection and GT at the time of recurrence after temozolomide/radiation therapy. The clinical outcome suggests a favorable safety profile, with no wound-related complications. OS was 20.0 months (~600 days) for unmethylated MGMT patients and 37.4 months (~1120 days) for methylated MGMT patients. The safety profile and potential efficacy signal warrant further investigation, particularly for MGMTm glioblastoma patients.

Under-estimating the complexity of a disease and unrestrained optimism of efficacy often result in premature termination in the clinical translation of potentially promising therapies. Brachytherapy, the placement of radioactive sources adjacent to cancerous tissues^1–3^ as a glioblastoma treatment, is a case in point. The pivotal observation that >80% of glioblastomas recur near the resection site^4–7^ focused research on local control, with the assumption that such control will translate into meaningful survival gains. While the initial brachytherapy studies showed promise^8^ and two randomized clinical trials (RCTs) demonstrated that brachytherapy improved glioblastoma local control,^9,10^ the less than impressive median survivals reported by these two RCTs decimated subsequent interests in brachytherapy.^9,10^ In retrospect, the reactions to these two studies seem draconian. Consider the following: the most impressive effect of the standard-of-care chemotherapy for glioblastoma, temozolomide, is not the two-month difference in median survival but the presence of a “tail” on the survival curve of the temozolomide treated patients—tails that were also observed in the brachytherapy studies.^11^ Moreover, the two RCTs were grossly underpowered if brachytherapy bears efficacy comparable to temozolomide or Optune®.^9–12^ Finally, as new glioblastoma therapeutic agents have emerged, the potential synergy between brachytherapy and these agents warrants investigation.

GT, or surgically targeted radiation therapy (STaRT), is a brachytherapy platform consisting of titanium encapsulated Cesium-131 seeds (^131^Cs, model CS-1, Rev2, IsoRay Medical Inc., Richland WA) embedded with an absorbable collagen matrix (Supplementary Figure 1). Each GT is 2 cm x 2 cm x 0.4 cm and contains four ^131^Cs radioactive seeds with a source strength of 3.5 U on the day of implant. The location and spacing of seeds within the collagen carrier were designed to optimize the dose delivered by the 30-keV characteristic x-ray photons emitted by ^131^Cs. The platform takes advantage of the surgeon’s familiarity with absorbable collagen sponges to facilitate the administration of brachytherapy implants. The low dose rate ^131^Cs seeds deliver 120–150 Gy at the matrix surface and 60–80 Gy at 5 mm depth, with rapid dose fall-off thereafter.^3,13^ The collagen matrix remains intact for approximately six weeks, maintaining the resection cavity architecture for approximately four half-lives of ^131^Cs (9.7 days) to prevent radiation “hot spots” associated with resection cavity collapse.^3^ GT received FDA clearance to treat recurrent brain tumors in late 2018.

Here, we report the first clinical series describing the use of GT since its approval in recurrent glioblastoma patients. Since patients with IDH mutated glioblastomas exhibit clinical courses that fundamentally differ from those with wild-type IDH (wtIDH),^14,15^ we restricted this study to wtIDH glioblastomas. In addition, we stratified our results based on MGMT promoter status, given the prognostic importance of this molecular biomarker in glioblastoma patients.^16,17^ Finally, all patients in this study cohort recurred after receiving standard of care, concurrent temozolomide/radiation therapy treatment.

## Materials and Methods

### Patient Population, Imaging Follow-Up, and Data Collection

The study was reviewed and approved by the University of Minnesota Human Research Protection Program. Data were prospectively collected from twenty-two consecutive patients with tissue confirmed recurrent glioblastoma who consented to and underwent GT placement between 2019 to 2020. The decision to offer GT placement was reviewed in a multi-disciplinary brain tumor board, consisting of representatives from neurosurgery, neuroradiology, neuro-oncology, radiation oncology, and neuropathology. The patient met with the radiation oncologist to review the recommended precautions for radiation safety. In accordance with the standard of care for our institution, magnetic resonance imaging (MRI) of the brain with and without contrast and clinical follow-up was conducted every 2–3 months (or earlier). Shorter interval MRI and clinical visits were instituted if imaging or clinical findings suggest recurrence.

Pathology findings were reviewed by two independent board-certified neuropathologists (WB and LC). MGMT promoter methylation and IDH mutation status were assessed at the time of initial diagnosis and recurrence. MGMT promoter methylation was evaluated using quantitative methylation-specific PCR as described previously.^18^ IDH mutation status was assessed by immunohistochemical staining and targeted DNA sequencing. No changes in MGMT promoter and IDH status were observed when comparing these results.

### GT Implant Preparation and Brachytherapy Dosimetry

Our institution’s commissioning and protocol for GT implementation were designed following the as low as reasonably achievable (ALARA) principle as described in a previous article.^13^

For postimplant treatment planning, one-millimeter thin-cut computed tomography (CT) and MRI (T1-weighted with/without gadolinium) of the brain were obtained within 24 hours of the procedure. The resection cavity was defined as the surface of the postresection surgical bed. High-risk clinical target volume (HR-CTV) was defined as regions including 5 mm expansion of the resection cavity abutting uninvolved brain parenchyma. A 60 Gray (Gy) dose was prescribed to the HR-CTV for all patients. Postoperative GT dosimetry parameters were evaluated, including HR-CTV D_90_ (dose received by 90% of the HR-CTV), HR-CTV V_50_ (volume receiving 50% (or 30 Gy) of the prescribed dose), HR-CTV V_100_ (volume receiving 100% (or 60 Gy) of the prescribed dose, and HR-CTV V_150_ (volume receiving 150% (or 90 Gy) of the prescribed dose.^19–21^

The medical physicist reviewed radiation safety aspects with the patient before patient discharge from the hospital, and the patient or patient’s caregiver signed a radiation safety release form.

### Neurosurgical Technique and Postoperative Assessment

All treated patients underwent 5-aminolevulinic acid (5-ALA, Gleolan, NX Development Corp., Lexington KY) guided resection followed by intraoperative MRI (Skyra intraoperative 3.0 T MRI, IMRIS, Minnetonka MN). If residual tumor was seen in an area considered safe, additional resection was performed. Once maximally safe resection was completed, and frozen pathology confirmed glioblastoma recurrence, GTs were implanted, covering the entire resection cavity. Incisions were closed with interrupted 2’0 Vicryl (Ethicon, Somerville, NJ) for the galea layer followed by 3’0 Monocryl skin closure (Ethicon, Somerville, NJ) and Exofin (Chemence Medical, Alpharetta, GA) application. For patients treated with bevacizumab, the surgery was performed a month after the last bevacizumab dose.

Postoperative care was carried out as per standard of care. The surgeon evaluated the patient around two weeks postprocedure, and the neuro-oncologist and the radiation oncologist within a month of surgery. The neuro-oncologist evaluated the patient every 2–3 months unless imaging or clinical findings warranted more frequent evaluation. There was no loss to follow-up.

### Imaging and Statistical Analysis

Surveillance MRIs were interpreted by the neuro-radiologist on duty and subsequently reviewed by an independent neuro-radiologist who reviews for the brain tumor board (MF, MG, or CO). Contouring of contrast-enhancing and T1 bright regions in the postoperative MRIs was performed by CF and independently confirmed by KD. PFS, including both local and distant recurrence, was determined based on the RANO criteria.^22^ Local control (LC) was defined as the absence of new T1 contrast-enhancing lesions within the HR-CTV volume on two sequential MRIs.

LC, OS, and PFS data were plotted using Prism 9 (Graphpad, San Diego, CA). Survival analysis was performed using Kaplan-Meier plots and Log-rank (Mantel-cox) test. Statistical significance is defined as *P* < 0.05. For group comparison, such as age, one-way ANOVA testing was performed with multi-comparison and Tukey correction.

## Results

### Patient Characteristics

The demographics of the 22 patients in the GT-implanted glioblastoma cohort were similar to previously reported glioblastoma series.^2,23–28^ The male:female ratio was 2:1, the cohort’s mean age was 57.7 years (range: 37–74), and the mean KPS of the cohort was 80 (range 60–100; Table 1). 18/22 (81.8%) of the patients presented with neurologic symptoms referrable to the glioblastoma recurrence. 14/22 (63.6%) underwent a single resection, and 8/22 (36.4%) underwent two prior resections. The mean time from the last surgery was 357 days (range: 122–970 days). All glioblastomas were IDH wild type. There were six MGMTm and 16 MGMTu glioblastomas. The mean time to repeat surgery and GT placement were significantly longer in the MGMTm patients relative to the MGMTu patients (Supplementary Figure 2).

**Table 1.** Patient Characteristics

Patient	Age	Sex (M/F)	Preprocedure KPS	Symptom	Steroid prior to procedure	Prior avastin	Time from last surgery (days)	Pathology	IDH	MGMT	Recurrence	Prior surgeries	Location of metastasis
1	50	F	80	left hemibody proprioceptive deficit	no	no	122	Glioblastoma	wt	u	3rd	2	right parietal
2	68	M	100	left arm weakness	no	no	360	Glioblastoma	wt	u	2nd	1	right motor
3	37	M	90	left hand discoordination	yes	no	197	Glioblastoma	wt	u	3rd	2	right temporal
3*	*	*	*	*	*	*	*	Glioblastoma	wt	u	*	*	right parietal
4	59	M	70	gait instability	no	yes	250	Glioblastoma	wt	m	3rd	2	right frontal
5	70	M	100	none	no	no	970	Glioblastoma	wt	m	2nd	1	right temporal
6	57	M	100	left hand discoordination	yes	no	416	Glioblastoma	wt	u	2nd	1	right parietal
7	49	M	60	moderate expressive aphasia, right hemiparesis	yes	yes	197	Glioblastoma	wt	u	3rd	2	left motor
8	74	F	70	gait instability	no	no	135	Glioblastoma	wt	u	2nd	1	right frontal
9	57	F	70	none	yes	no	215	Gliosarcoma	wt	u	2nd	1	left frontal
10	49	M	70	expressive aphasia	yes	no	902	Glioblastoma	wt	u	2nd	1	left frontal
11	73	M	70	left homonymous hemianopsia	yes	no	127	Glioblastoma	wt	u	2nd	1	right temporal/occipital
12	58	F	70	gait instability	yes	no	226	Glioblastoma	wt	u	2nd	1	right frontal
13	52	M	100	none	no	no	158	Glioblastoma	wt	u	3rd	2	right temporal
14	57	M	100	left hand discoordination	no	no	288	Glioblastoma	wt	u	2nd	1	right parietal
15	60	M	70	left hemiapresis	no	no	220	Glioblastoma	wt	u	2nd	1	right motor
16	73	F	70	right hemonymous hemianopsia	no	no	612	Glioblastoma	wt	m	2nd	1	left occipital
17	37	M	100	none	no	no	253	Glioblastoma	wt	u	3rd	2	right temporal
18	62	M	100	gait instability	no	no	890	Glioblastoma	wt	m	2nd	1	right frontal
19	45	M	80	right hand discoordination	no	no	612	Glioblastoma	wt	m	2nd	1	right temporal
20	57	M	70	gait instability	yes	no	231	Glioblastoma	wt	u	2nd	1	left motor
21	65	F	60	left homonymous hemianopsia	yes	no	477	Glioblastoma	wt	m	3rd	2	right occipital
22	62	F	70	expressive aphasia	no	no	701	Glioblastoma	wt	m	3rd	2	left temporal
**Average**	**57.77**		**80.45**				**389.05**					**1.36**	

*Two lesions treated in the same patient.

NA: not applicable.

In terms of oncologic treatment, all patients underwent standard of care temozolomide-radiation therapy at the time of initial diagnosis. Lomustine salvage therapy was used in 20/22 patients (91%) in the GT treatment cohort at recurrence. Bevacizumab was reserved until symptomatic decline or time of palliation. Only two patients in this series (patients 4 and 7) required bevacizumab prior to GT implant (Supplementary Table 1). Patients who recurred within six months of temozolomide-radiation therapy were not considered for GT placement to minimize the risk of radiation toxicity.

All surgical procedures were performed by the senior author (CCC). 4/22 (18.1%) of the patients showed residual tumor on intraoperative MRI after 5-ALA-guided surgery. Two of these patients underwent additional surgical removal after the intraoperative MRI to achieve gross total resection. Two patients (patients 7 and 10) underwent awake craniotomies, and resection was terminated due to involvement of functional cortex identified during motor mapping, resulting in subtotal resections (Table 2). Apart from these patients, any residual contrast enhancement seen on the postoperative T1 MRI with gadolinium (Table 2) was also seen on the postoperative T1 MRI without gadolinium, suggesting that these residual contrast enhancements were due to postoperative changes rather than residual tumor. In this context, 20/22 (90.9%) of the patients in this study cohort underwent gross total resections.

**Table 2.** GammaTile Resection and Dosimetric Parameters

Patient	Lesion	Resection cavity volume (postop resection cavity) (cm^3^)	Number of tiles implanted	PostOp Enhancement on T1 MRI without gadolinium (cm^3^)	PostOp Enhancement on MRI T1 with gadolinium volume (cm^3^)	High-risk clinical target volume (HR-CTV) (cm^3^)	HR-CTV D_90_ - Dose received by 90% of the volume (Gy)	HR-CTV V_50_ - Volume receiving 50% (30Gy) of the prescribed dose (%)	HR-CTV V_100_ - Volume receiving 100% (60Gy) of the prescribed dose (%)	HR-CTV V_150_ - Volume receiving 150% (90Gy) of the prescribed dose (%)
1	1	16.4	5	9.2	9.2	43.1	46.05	99.93	73.00	45.84
2	2	5.8	3.5	2.1	2.1	14.3	56.04	100.00	86.69	51.37
3*	3	15.4	3	0	0	19.2	42.73	99.32	72.10	38.58
3*	4	43.1	6	0	0	25.3	37.89	98.48	41.94	14.50
4	5	16.4	6	2.8	2.8	21.8	66.76	100.00	96.70	53.49
5	6	35.2	6	0	0	11.1	58.82	99.99	88.85	56.45
6	7	6.4	3.5	1.7	1.7	15.4	41.58	98.33	72.51	43.76
7	8	17.3	6	4.8	28.9	23.6	63.39	100.00	92.89	51.17
8	9	19.5	6	1.2	1.2	26	38.12	95.92	68.19	34.14
9	10	78.6	12	0.6	0.6	58.3	44.05	95.28	77.96	43.99
10	11	27.3	7.5	3.7	21.6	45.1	41.97	99.02	68.17	34.73
11	12	19.5	16	1.6	1.6	55.8	98.66	100.00	98.86	93.03
12	13	8.4	6	2.3	2.3	29.9	76.60	100.00	97.15	79.58
13	14	12.5	6	0.4	0.4	18.8	70.96	100.00	96.28	72.77
14	15	13.2	6	0.6	0.6	35.6	66.78	100.00	94.18	72.67
15	16	5.2	4	0.6	0.6	14.1	59.62	99.80	89.64	58.69
16	17	18.9	6	0.2	0.2	25.7	56.92	100.00	85.59	41.43
17	18	27.7	6	0.2	0.2	22.1	48.75	99.18	72.73	27.64
18	19	45.1	10	0	0	39	62.94	100.00	93.16	45.67
19	20	5.7	4	0	0	11.6	38.16	95.91	74.34	50.86
20	21	4.8	5	1.3	1.3	9.6	92.33	100.00	99.33	91.34
21	22	53.2	6	0.8	0.8	29	31.73	91.13	57.74	24.53
22	23	14.5	6	0	0	16.9	70.72	100.00	96.25	68.03
**Average**		**22.18**	**6.33**	**1.48**	**3.31**	**26.58**	**57.02**	**98.80**	**82.36**	**51.92**

*Two lesions treated in the same patient.

### GT Placement and Dosimetry

The average resection cavity volume was 22.18 cm^3^ (range: 4.8-78.6). The median number of GT units implanted was 6 (range: 3 to 16). In all cases, the HR-CTV was prescribed 60 Gy. The average HR-CTV D_90_ was 57.0 Gy (range: 31.7–98.7 Gy). The average HR-CTV V_50_, HR-CTV V_100_, and HR-CTV V_150_ were: 98.8 % (range: 91.1–100.0%), 82.4% (range: 41.9–99.3%), and 51.9% (range: 14.5–93.0%) respectively (Table 2).

### Hospital Course, Re-Admission, and Safety

All patients were evaluated by physical and occupational therapy before discharged home. As seen in Supplementary Table 2, 18/22 (81.8%) of the patients were discharged home before or on postoperative day 3. Of the four patients who were not discharged prior to postoperative 3, two patients required extended stay due to rehabilitation placement (patients 1 and 9), and two patients required prolonged hospitalization because of postoperative complications (patients 4 and 5). The clinical course of these two patients is described in more detail in the complications section.

There was one re-admission within 30 days for cerebrospinal fluid (CSF) leak, requiring antibiotics treatment, wound suture reinforcement, and a 5-day course of lumbar drain. The leak resolved subsequently, and the patient was discharged home. However, the patient developed a sizable, symptomatic pseudo-meningocele that ultimately required the placement of a ventriculoperitoneal shunt. This clinical course suggests non-obstructive hydrocephalus as a contributing cause of the CSF leak. There were no other wound complications in the GT-treated patients.

There were no new neurologic deficits that developed after GT placement. However, 6/22 (27.2%) patients suffered a decline in KPS secondary to pre-existing deficits. Of note, the KPS declines did not negatively impact the physical therapist/occupational therapist assessment in terms of safety for discharge to home (Supplementary Table 2), and the patients recovered from these declines by the one-month follow-up.

### Local Control and Overall Survival

The clinical practice at our institution is to generally reserve bevacizumab until needed for symptomatic decline or the time of palliation. This practice preference allowed an opportunity to assess local control without the confounding effects of bevacizumab. That said, two patients (patients 4 and 7) were treated with bevacizumab prior to surgery and resumed treatment after GT placement (Supplementary Table 1). In both patients, however, new contrast enhancements emerged at the time of recurrence.

**Figure 1.**
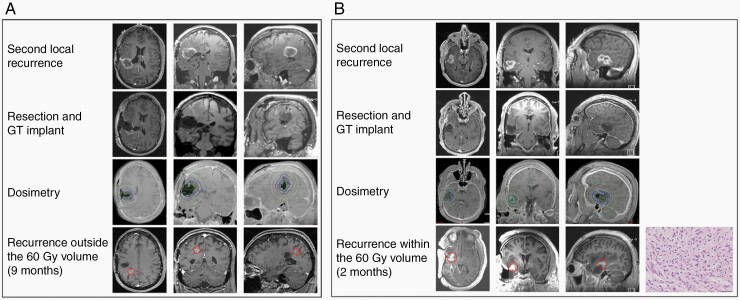
Illustrative examples of GammaTile® (GT) dosimetry and local control. (A) Patient 1 developed new contrast enhancement outside of the HR-CTV (See Methods). Preoperative axial, coronal, and sagittal MR postgadolinium T1 images (top row) and corresponding postoperative images (second row) are shown. GT dosimetry is shown on the third row. The green line indicates the HR-CTV. Axial, coronal, and sagittal MR postgadolinium T1 images at the time of recurrence are shown in the fourth row. (B) Patient 13 developed new contrast enhancement within the HR-CTV. The arrangement of images is described in (A). Hematoxylin and eosin (H&E) staining of the clinical specimen derived from resection of the contrast-enhancing lesion is shown in the rightmost panel on the fourth row. Pathology review of the sample revealed the presence of active tumor, indicating tumor recurrence.

**Figure 2.**
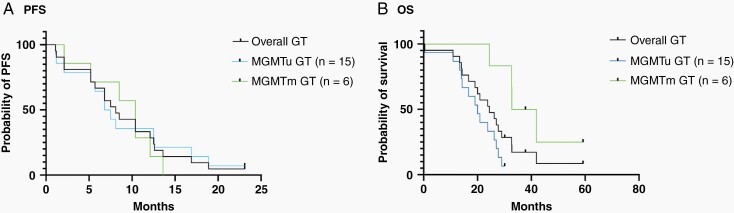
Assessment of progression-free survival (PFS) and overall survival (OS) after GT implant. The Kaplan-Meier analysis for (A) PFS for all GT-treated patients, GT-treated MGMTu patients, and GT-treated MGMTm patients. (B) OS for all GT-treated patients, GT-treated MGMTu patients, and GT-treated MGMTm patients.

A typical distant recurrence of new contrast enhancement outside of the HR-CTV volume is shown in Figure 1A. A recurrence within the HR-CTV volume is shown in Figure 1B. The local control of MGMTm and MGMTu appeared comparable in this study cohort. As such, we combined MGMTm and MGMTu patients to provide an estimate for the PFS. The median PFS for the entire cohort was 8.1 months (Figure 2A). The local control at 6 and 12 months were 86 and 81%, respectively. We were unable to identify correlations between the brachytherapy dosimetric parameters (including HR-CTV D_90_, HR-CTV V_50_, HR-CTV V_100_, and HR-CTV V_150_) and the likelihood of PFS (Supplementary Figure 3A–D). However, it is notable that the GT-treated patient (patients 7) who most rapidly progressed locally after surgery harbored residual tumor volumes extending beyond the HR-CTV (Supplementary Figure 4).

The median survival for the overall GT-treated cohort was 24.4 months (Figure 2B). MGMT promoter methylation status was correlated with improved OS in the GT-implanted patients (Figure 2B). The median survival for GT-treated MGMTm patients was 37.4 months. The median survival of GT-treated MGMTu glioblastoma patients was 20.0 months (Figure 2B).

### Complications

**Figure 3.**
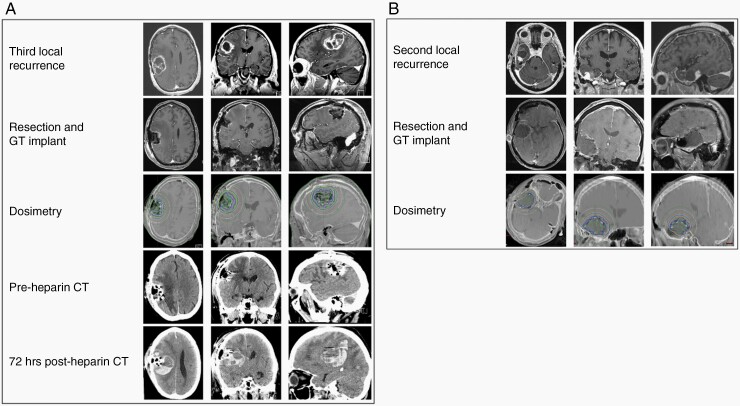
Postoperative morbidity in GT-treated patients. (A) Patient 4 (history of lower extremity DVT receiving Coumadin prior to surgery) required emergent thrombolysis followed by full heparinization for an ischemic limb on postoperative day three and suffered neurologic decline two days after full anticoagulation. Top panel: preoperative axial, coronal, and sagittal MR postgadolinium T1 images. Second panel: corresponding postoperative images. Third panel: GT dosimetry. Fourth panel: preheparization axial, coronal, and sagittal CT. Fifth pane: postheparization axial, coronal, and sagittal CT. (B) Patient 5 emerged from surgical resection and GT placement with a non-focal examination (preoperative gadolinium enhanced MRI, postoperative gadolinium enhanced MRI, and GT dosimetry are shown in rows one through three) but suffered a new-onset seizure on postoperative day one. He recovered from these seizures without deficits.

There were two complications in this cohort. First, patient 4 (MGMTu) had a prior history of lower extremity deep vein thrombosis (DVT) and was on Coumadin prior to surgery. While he emerged from the surgical resection neurologically similar to his preoperative condition, he developed an ischemic limb on postoperative day three, requiring emergent thrombolysis followed by full heparinization. Unfortunately, the patient suffered neurologic decline two days after full anticoagulation was achieved, and emergent CT showed a sizable acute intracranial hemorrhage with significant mass effect (Figure 3A). After a difficult discussion, the family opted for transition to comfort measures, and the patient died on postoperative day 10.

Patient 5 (MGMTm) emerged from the surgery neurologically non-focal but subsequently suffered a new-onset seizure on postoperative day one. Later, the patient recovered fully from the seizure and was discharged home on postoperative day 15 (Figure 3B).

### Radiation Necrosis

Given the short range of GT brachytherapy, radiation necrosis is most likely to occur within the HR-CTV region. Of the GT-treated patients, new contrast enhancement/FLAIR in this region was seen in five patients. Four patients (patients 2, 5, 7, and 9) underwent subsequent resection, with pathology confirming tumor recurrence. Patient 7 had residual tumor volume in the anterior margin that extended beyond HR-CTV. On brain MRI taken a month after resection/GT placement, significant expansion of the anterior contrast-enhancing region was seen (Supplementary Figure 4). MR perfusion imaging demonstrated increased perfusion in this enhancing region. These findings indicate tumor progression for patient 7. Given these results, we believed that none of the treated patients suffered from radiation necrosis.^29,30^

### A Control Cohort of Patients Underwent Gross Total Resection During the Same Study Period Without GT Placement

We wished to compare the outcomes of this cohort to those reported in the published literature. However, we were unable to identify surgical outcomes after repeat resection for patients with both IDH and MGMT promoter status. Therefore, we identified patients with unifocal wt-IDH glioblastoma who underwent gross total resection of recurrent glioblastoma at our institution by the senior author (CCC) during the same study period. All patients in the control cohort underwent standard of care temozolomide/radiation therapy at the time of initial diagnosis. These patients declined GT or were enrolled in clinical trials that did not allow GT placement at recurrence. We identified a total of 21 such patients (16 MGMTu and five MGMTm) with recurrent glioblastoma. The demographic data and the treatment history of this control cohort are shown in Supplementary Tables 3 and 4.

As with the GT-treated cohort, Lomustine was prescribed as salvage therapy at recurrence for 18/21 patients (86%) in the control cohort. Bevacizumab was reserved until needed for symptomatic decline or the time of palliation. While this “control” cohort was not matched at a patient level to the GT-treated cohort, the proportion of patients receiving immunotherapy, targeted therapy, or participating in clinical trials at the time of recurrence were comparable (Supplementary Tables 1 and 3). Similarly, the age, KPS, and the number of prior surgeries were comparable between the “control” and the GT-treated cohort (Supplementary Figure 5).

**Figure 4.**
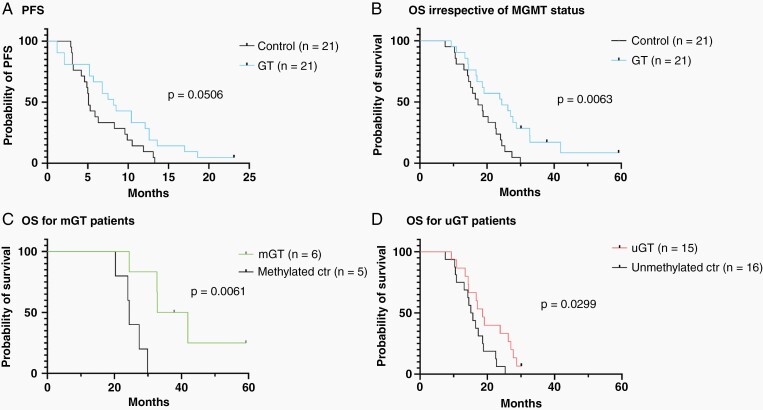
PFS and OS between the GT-treated and a “control” cohort who underwent re-resection without GT. Shown are Kaplan-Meier analysis for (A) PFS for the control and GT-treated cohorts without stratification by MGMT promoter methylation status. (B) OS for the control and GT-treated cohorts. (C) OS for the control and GT-treated MGMTm (mGT) glioblastoma patients. (D) OS for the control and GT-treated MGMTu (uGT) glioblastoma patients.

The median PFS for the GT-treated patients was lengthened relative to the control cohort (8.2 versus 5.1 months) but did not reach statistical significance (*P* = 0.0506; Figure 4A). The OS of the entire control cohort (without consideration for MGMT status) was shorter (17 months) than the overall GT-treated cohort (24.4 months, *P* = 0.0063; Figure 4B). The OS for the GT-treated MGMTm group was 37.3 months and improved relative to the MGMTm control group (24.4 months; *P* < 0.0061, Figure 4C). The OS of the control MGMTu cohort was 15 months, which was shorter than that observed for the GT-treated MGMTu cohort (18.6 months, *P* = 0.0299; Figure 4D).

Overall, these results support further investigation of GT as an adjunctive treatment option for recurrent glioblastoma patients who are deemed candidates for repeat surgical resection.

## Discussion

Relative to all currently available glioblastoma therapies, radiation is considered one of the most effective.^11,29,30^ However, its efficacy is limited by the pliability of glioblastoma cell states in radiation response and the multiplicity of resistance mechanisms.^31–34^ The efficacy of brachytherapy should be considered in this context. It may not be the “magic bullet” that many previous generations of brachytherapy advocates envisioned. However, brachytherapy offers dose-escalation that conformed to the resection cavity without delay related to surgical recovery. In this study, the safety profile of GT following maximal safe resection was comparable to published surgical series of glioblastoma re-resection.^35^ Despite a cohort of patients who underwent second and third resection through the same surgical incision, no wound infections or dehiscence was observed in this cohort. The one CSF leak occurred in a patient who ultimately required ventriculoperitoneal shunting, suggesting hydrocephalus as the etiology. The lengths of hospital stay and operative morbidity/mortality for the GT-treated cohort were comparable to those reported for glioblastoma patients who underwent re-resection without GT implant.^36,37^ The observation that 27% of the patients in this cohort suffered KPS decline after surgery due to worsening existing deficits was comparable with the reported incidence of 16–40% postoperative morbidity after re-resection.^35,38,39^ Importantly, none of the patients in this cohort suffered from adverse radiation effects that required medical or surgical intervention.

While there is a sizable literature examining clinical outcomes after glioblastoma re-resection at the time of recurrence,^35^ the lack of IDH and MGMT promotor methylation status in these studies rendered comparisons difficult. For instance, the presence of IDH mutated or over-representation of MGMTm glioblastomas in any cohort will “inflate” the OS.^28,40^ In this context, 71.4% of this cohort consisted of the prognostically unfavorable MGMTu glioblastomas, which is over-represented relative to the expected frequency of ~55 % MGMTu.^28^ Despite the over-representation of these prognostically unfavorable patients, the PFS (8.2 months) and OS (24.4 months) of the entire cohort (irrespective of MGMT promoter methylation status) compared favorably to previously published series of recurrent glioblastoma patients who underwent re-resection,^35^ where PFS ranged 3.75–9.37 months and OS ranged 15–27 months.

The 16–40% postoperative neurologic decline renders surgical resection a daunting proposition for recurrent glioblastoma patients. On the other hand, patient’s progressive decline is expected secondary to tumor-related mass effects. While repeat external beam radiation therapy is an option for recurrent glioblastomas, it is primarily a palliative measure without meaningful likelihood for functional restoration or durable functional preservation. In contrast, minor declines are observed in some patients in the immediate postoperative setting, improvements are typically seen by the one-month follow-up unless the patient suffered a surgical adverse event.

Despite our limited sample size, our results suggest that MGMTm glioblastoma patients derive greater survival benefits from GT. The finding is consistent with Brandes et al. that MGMTm glioblastoma is more likely to recur locally than their MGMTu counterpart.^41^ GT implant likely suppresses such local recurrence within the HR-CTV to improve clinical survival. Since GT-treated MGMTm glioblastoma patients ultimately suffer distant recurrences, effective therapy will require strategies that mitigate the likelihood of such recurrences.

From a psychological perspective, the devastating impact of local recurrence on the patient should not be underestimated, especially when the recurrence is in close temporal proximity to the initial surgery. Frequently, local recurrences trigger discussion of palliation. As such, achieving local control can potentially delay the decision for palliation and extend the time crucial for select classes of therapeutics, such as immunotherapy.^42,43^ This consideration is important in interpreting the GT-associated efficacy signals.^44,45^

As a prospective case series, the study suffers from limitations inherent within the study design.^46,47^ The limited sample size prohibited meaningful interpretation of potential dose-response relationships between brachytherapy dose and local control, especially given the heterogeneity in radiation resistance mechanism for glioblastomas.^31–34^ That said, the GT-treated patients who most rapidly progressed locally after surgery (patients 7 progressed 35 days after GT implant) harbored residual tumor volumes extending beyond the HR-CTV (Supplementary Figure 4), suggesting the importance of gross total resection. Additionally, while the study’s sample size is limited, it is within publication standards for pilot series.^48^ Given that GT has been FDA cleared for brain tumor treatment since 2018 and no clinical GT-treated glioblastoma patient data are available, we believe that the timing of this study achieves a reasonable balance between the meaningful follow-up period and the timely sharing of clinical data for a newly available therapeutic.

Another limitation of the study relates to the generalization of the presented results. The patients who elected to undergo GT placement tend to be motivated to seek care beyond the standard of care. In addition, the exclusion of glioblastoma patients who recurred within six months of standard of care radiation therapy also enriched for prognostically favorable patients. On the other hand, other aspects of this study over-represented prognostically unfavorable patients, including a higher proportion of MGMTu patients and patients with symptomatic progression secondary to mass effect. The overall balance of these biases in survival prognostication is challenging to assess. Moreover, it is difficult to tease out the survival contribution of GT relative to other therapies that the patients received. Finally, while no brachytherapy-related adverse events are reported in this GT-treated cohort, the absence of the quality-of-life data renders it challenging to assess the overall impact of the therapy on the treated patient. To address this matter, a quality-of-life registry is recruiting GT-treated brain tumor patients.

(NCT04427384;https://clinicaltrials.gov/ct2/show/NCT04427384?term=gammatile&cond=brain+tumor&draw=2&rank=1).

Current options for recurrent glioblastoma include clinical trial participation, surgical resection, “second-line” chemotherapy, external beam radiation with or without bevacizumab, immunotherapy, targeted therapy based on genomic profiling, and Optune. The general principle guiding repeat resection for patients in this series include: 1) symptomatic progression, 2) lesion causing mass effect, 3) resection can be achieved without unacceptable morbidity risk, and 4) reasonable neurologic function (generally KPS > 70) at recurrence. Despite potential biases introduced by these selection criteria, our results suggest that brachytherapy may be of potential benefit for glioblastoma patients who undergo re-resection.

## Conclusion

Here we report the outcome of the first clinical series utilizing commercial GT since its FDA clearance in a cohort of recurrent glioblastoma patients. The exploratory safety and outcome data are favorable and support future investigations of GT as a component of a multi-modality treatment strategy for recurrent glioblastomas, particularly for MGMTm glioblastomas.

## Supplementary Material

vdab185_suppl_Supplementary_Material

vdab185_suppl_Supplementary_Legends
