# Supplementary material for: GammaTile® brachytherapy in the treatment of recurrent glioblastomas
Source: Neurooncol Adv. 2021 Dec 27;4(1):vdab185. doi: 10.1093/noajnl/vdab185 (PMC8788013; doi:10.1093/noajnl/vdab185)

**Supplemental Table 1.** Treatment regimen for GT treatment prior and after GT placement.

| **Patient #** | **Pre-GT** | **Post-GT** |
| --- | --- | --- |
| 1 | TMZ, RT, Toca511 | Ad-RTS-hIL12, Nivolumab, Bevacizumab |
| 2 | TMZ, RT, Toca511, Lomustine | Ad-RTS-hIL12, Nivolumab, Bevacizumab |
| 3* | TMZ, RT, ABT 414, Ad-RTS-hIL12 | Pembrolizumab, Bevacizumab |
| 4 | TMZ, RT, Lomustine, Bevacizumab | Pembrolizumab, Bevacizumab |
| 5 | TMZ, RT | Lomustine |
| 6 | TMZ, RT | Lomustine, Bevacizumab |
| 7 | TMZ, RT, Lomustine, Bevacizumab | Avastin , Everolimus |
| 8 | TMZ, RT | Lomustine, Bevacizumab |
| 9 | TMZ, RT | Lomustine |
| 10 | TMZ, RT | Lomustine, Bevacizumab |
| 11 | TMZ, RT | Lomustine, Bevacizumab |
| 12 | TMZ, RT | Lomustine, Bevacizumab |
| 13 | TMZ, RT, Lomustine | Bevacizumab |
| 14 | TMZ, RT, Atezolizumab | Lomustine, Ad-RTS-hIL12, Nivolumab, Bevacizumab |
| 15 | TMZ, RT | Lomustine, Bevacizumab |
| 16 | TMZ, RT, Lomustine | Bevacizumab |
| 17 | TMZ, RT, Lomustine | Pembrolizumab, Bevacizumab |
| 18 | TMZ, RT | Lomustine, Bevacizumab |
| 19 | TMZ, RT | Lomustine, Bevacizumab |
| 20 | TMZ, RT | Lomustine, Bevacizumab |
| 21 | TMZ, RT, Lomustine | Bevacizumab |
| 22 | TMZ, RT, Lomustine | Bevacizumab |

| *Toca511 was administered through NCT02414165 participation |
| --- |
| **Ad-RTS-hIL12 was administered through NCT03636477 participation or a Ziopharm sponsored EAP |
| ***ABT-414 was administered through NCT01800695 participation |
| ****Atezolizumab was administered through NCT03174197 participation |
|  |

**Supplemental Table 2.** Post-operative patient characteristics and complications.

| **Patient** | **Duration (days) follow-up** | **Post-operative Neurologic deficit** | **Post-surgery KPS** | **Hospital stay (days)** | **Readmission within 30 days** | **Wound breakdown** |
| --- | --- | --- | --- | --- | --- | --- |
| 1 | 279 | worsened proprioceptive deficit | 80 | 7 | no | no |
| 2 | 356 | None | 100 | 1 | no | no |
| 3 | 111 | None | 90 | 1 | no | no |
| 3* | 72 | None | 90 | 1 | no | no |
| 4 | 10 | Intracranial hemorrhage | 70 | 10 | NA | no |
| 5 | N/A | Post-operative seizure | 100 | 9 | no | no |
| 6 | 394 | worsened hemisensory deficit | 90 | 1 | no | no |
| 7 | 280 | None | 60 | 1 | no | no |
| 8 | 282 | None | 70 | 3 | no | no |
| 9 | 114 | None | 50 | 15 | no | no |
| 10 | N/A | None | 70 | 1 | no | no |
| 11 | 305 | worsened left homonymous hemianopsia | 70 | 2 | no | no |
| 12 | 819 | none | 70 | 1 | no | no |
| 13 | 250 | none | 100 | 1 | yes, CSF leak, resolved with LD | no |
| 14 | 499 | none | 100 | 1 | no | no |
| 15 | 628 | none | 70 | 1 | no | no |
| 16 | N/A | worsened right homonyous hemianopsia | 60 | 2 | no | no |
| 17 | 321 | none | 100 | 1 | no | no |
| 18 | 377 | none | 70 | 3 | no | no |
| 19 | 370 | none | 80 | 1 | no | no |
| 20 | 198 | worseed right hemi-paresis | 60 | 3 | no | no |
| 21 | 257 | worsened left hemibody strength | 60 | 3 | no | no |
| 22 | 284 | none | 70 | 2 | no | no |

*Two lesions treated in the same patient

**Supplemental Table 3.** Treatment regimen for control patients prior and after resection only.

| **Patient #** | | **PreOP** | **PostOP** |
| --- | --- | --- | --- |
| 1 | | TMZ, RT | Lomustine |
| 2 | | TMZ, RT, Lomustine | Bevacizumab, Pembrolizumab |
| 3 | | TMZ, RT | Lomustine |
| 4 | | TMZ, RT | Lomustine, Bevacizumab |
| 5 | | TMZ, RT, ABT414 | Lomustine, Bevacizumab |
| 6 | | TMZ, RT | Ad-RTS-hIL12, Nivolumab, Bevacizumab |
| 7 | | TMZ, RT, Lomustine | Pembrolizumab, Bevacizumab |
| 8 | | TMZ, RT | Lomustine, Bevacizumab |
| 9 | | TMZ, RT | Bevacizumab |
| 10 | | TMZ, RT | Lomustine, Bevacizumab |
| 11 | | TMZ, RT | Lomustine, Bevacizumab |
| 12 | | TMZ, RT, Lomustine, Bevacizumab | Pembrolizumab, Bevacizumab |
| 13 | | TMZ, RT | Lomustine, Bevacizumab |
| 14 | | TMZ, RT | Bevacizumab |
| 15 | | TMZ, RT | Lomustine, Bevacizumab |
| 16 | | TMZ, RT, Lomustine | Pembrolizumab, Bevacizumab |
| 17 | | TMZ, RT | Lomustine, Bevacizumab |
| 18 | | TMZ, RT | Lomustine |
| 19 | | TMZ, RT | Lomustine, Bevacizumab |
| 20 | | TMZ, RT | Lomustine, Bevacizumab |
| 21 | | TMZ, RT, Lomustine | Everolimus, Bevacizumab |
| *Ad-RTS-hIL12 was administered through NCT03636477 participation or a Ziopharm sponsored EAP | | | |
| **ABT-414 was administered through NCT01800695 participation | | | |
| ***For the GT cohort, the patient number matches Table 1. For the control cohort, patient 1-5 were MGMTm and the remaining patients were MGMTu | | | |

**Supplemental Table 4.** Demographics control patient cohort. MGMT methylated and MGMT unmethylated.

**MGMTm**

| **Patient** | **Age** | **Sex** | **Symptoms** | **KPS** | **PFS** |
| --- | --- | --- | --- | --- | --- |
| 1 | 70 | M | hemiparesis | 70 | 188 |
| 2 | 39 | F |  | 100 | 152 |
| 3 | 65 | M |  | 100 | 392 |
| 4 | 50 | F | cognitive decline | 80 | 176 |
| 5 | 68 | F |  | 100 | 400 |
| **Average** | **57** |  |  | **91** | **343** |

**MGMTu**

| **Patient** | **Age** | **Sex** | **Symptoms** | **KPS** | **PFS** |
| --- | --- | --- | --- | --- | --- |
| 1 | 59 | F | Expressive aphasia, right hemiparesis | 80 | 87 |
| 2 | 54 | M | Expressive/receptive aphasia, right homonymous hemianopsia | 70 | 298 |
| 3 | 59 | M | Gait instability | 70 | 139 |
| 4 | 62 | F | Expressive aphasia | 60 | 154 |
| 5 | 52 | F | Expressive aphasia | 90 | 248 |
| 6 | 68 | F | Left homonymous quadrantanopia | 80 | 92 |
| 7 | 68 | F | Expressive aphasia | 80 | 315 |
| 8 | 65 | F | Confusion | 70 | 93 |
| 9 | 63 | M | Left hand discoordination | 70 | 147 |
| 10 | 29 | F | Compromised short term memory | 70 | 158 |
| 11 | 59 | M | Right hemiparesis | 50 | 96 |
| 12 | 29 | M | Confusion | 50 | 89 |
| 13 | 87 | F | Confusion | 60 | 125 |
| 14 | 67 | F | Headache | 70 | 150 |
| 15 | 64 | F | right quadranopia, facial droop | 50 | 289 |
| 16 | 25 | M | None | 100 | 357 |
| **Average** | **56** |  |  | **70** | **177** |


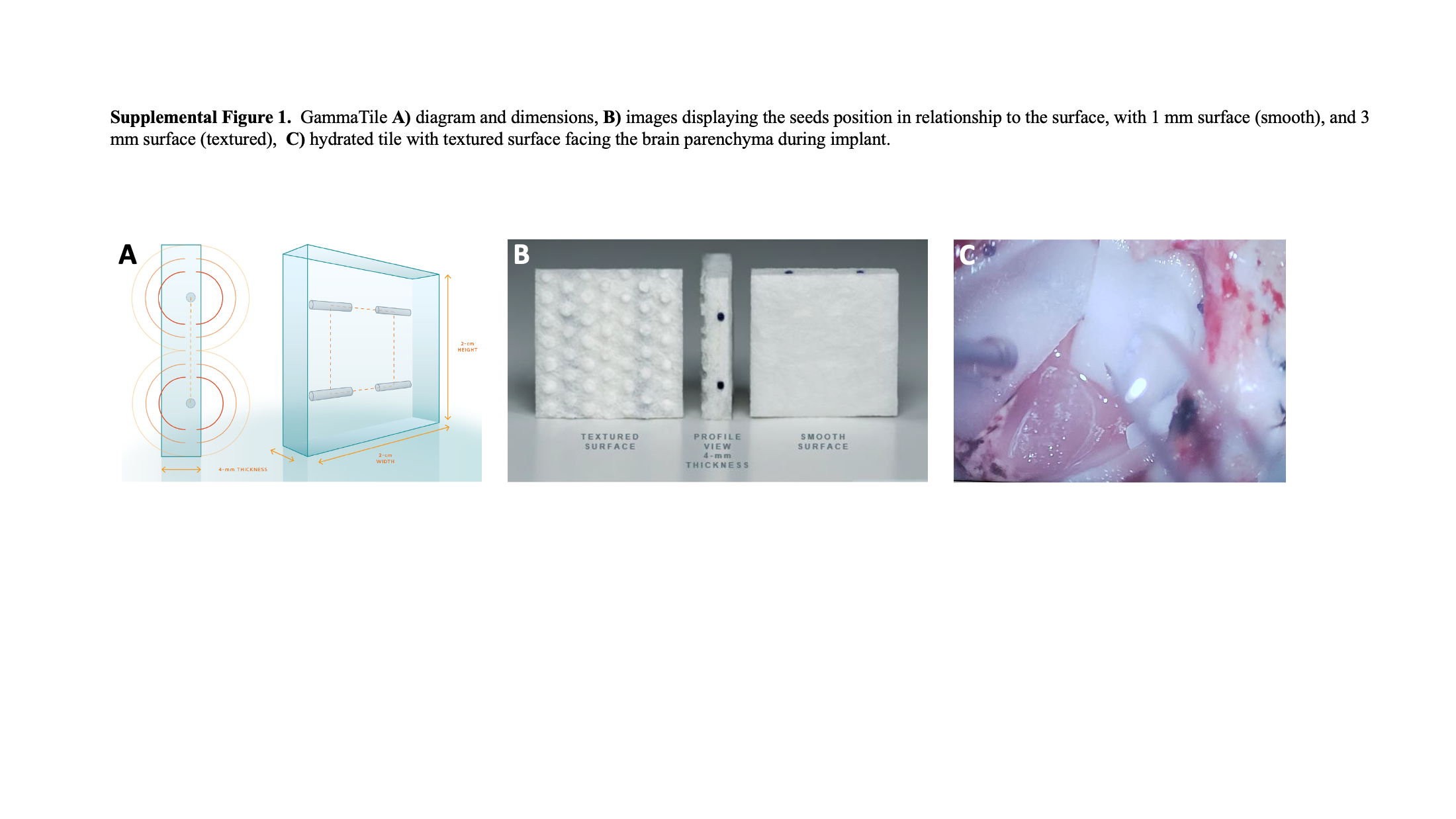


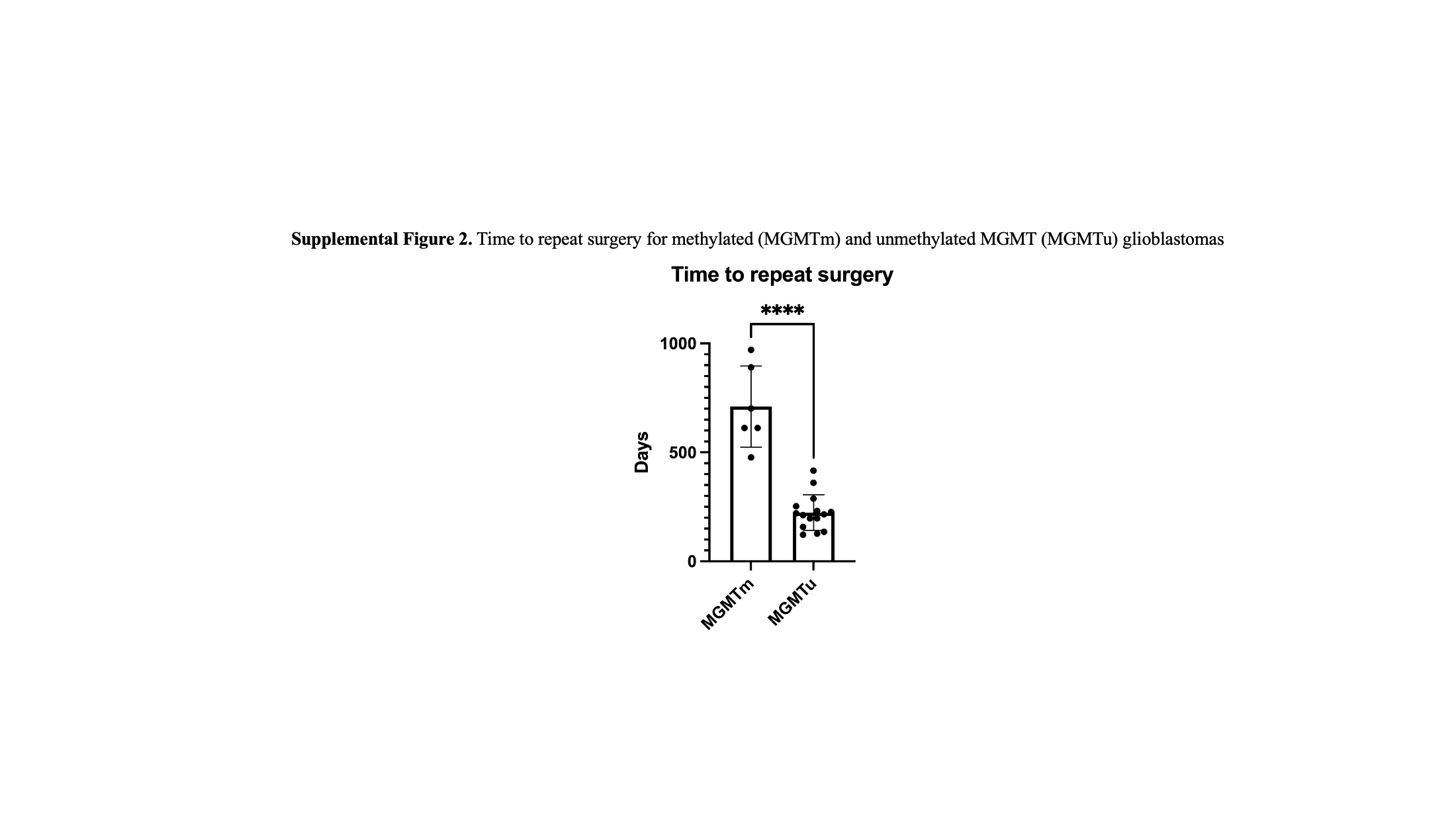


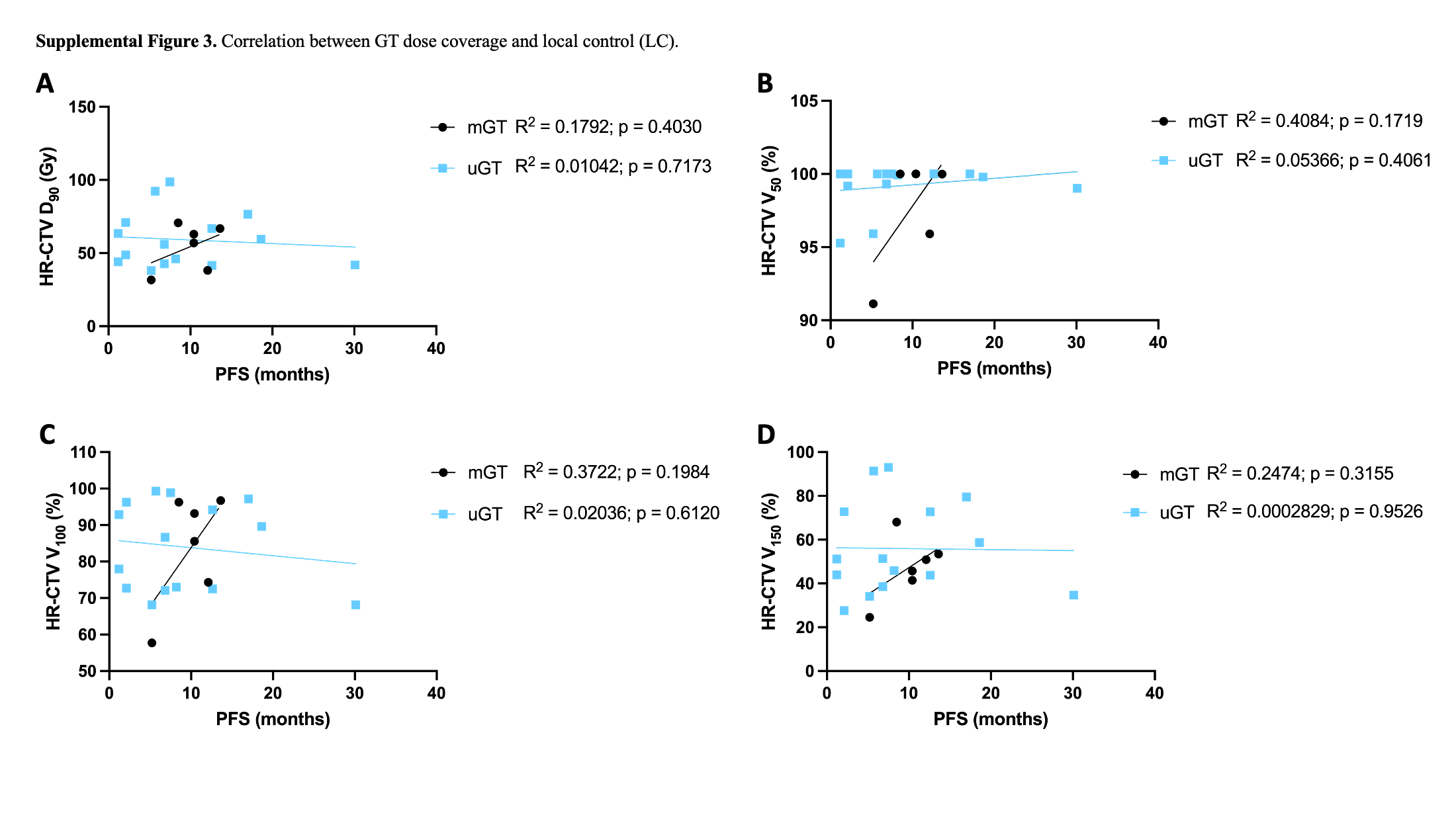


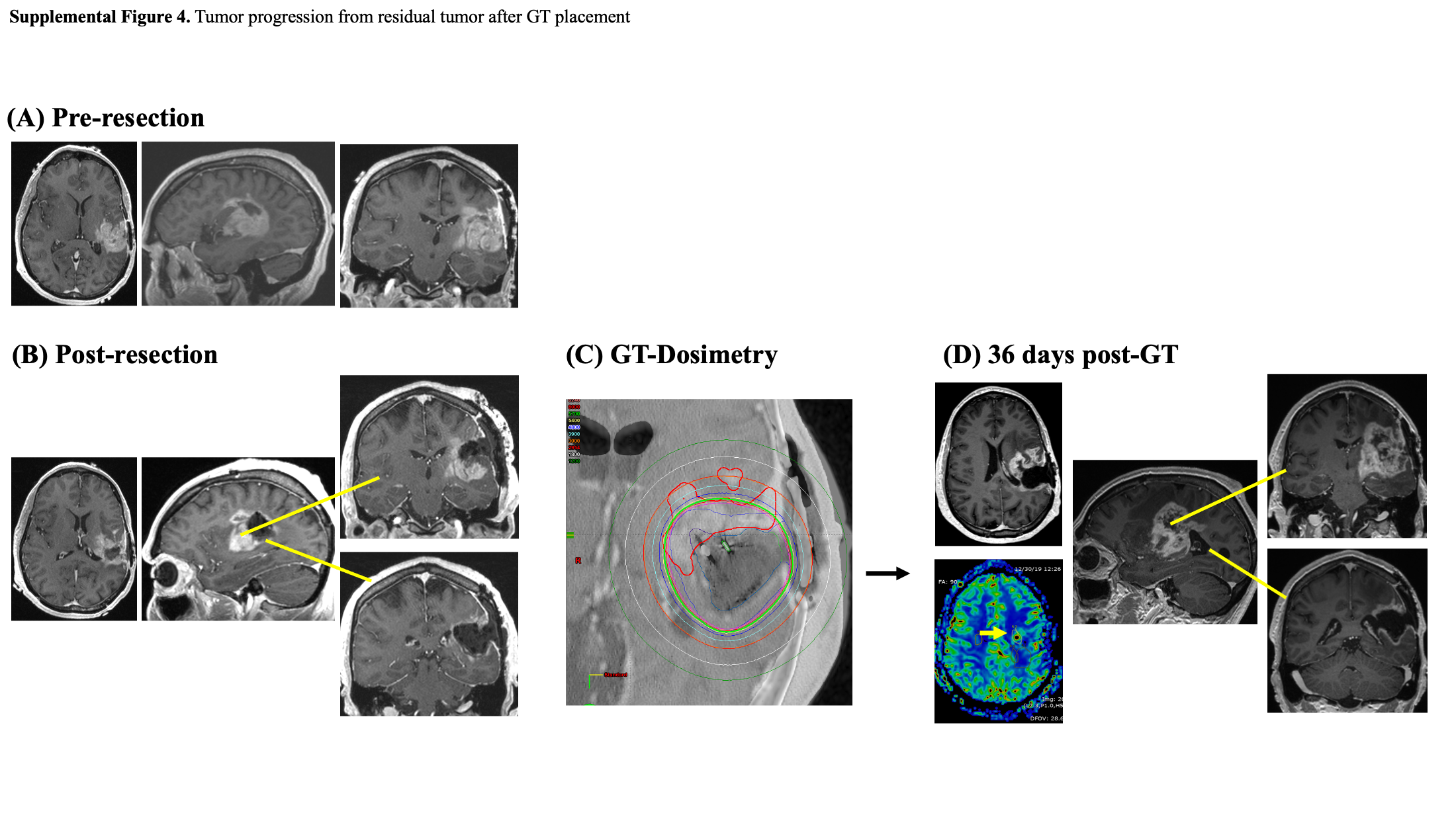


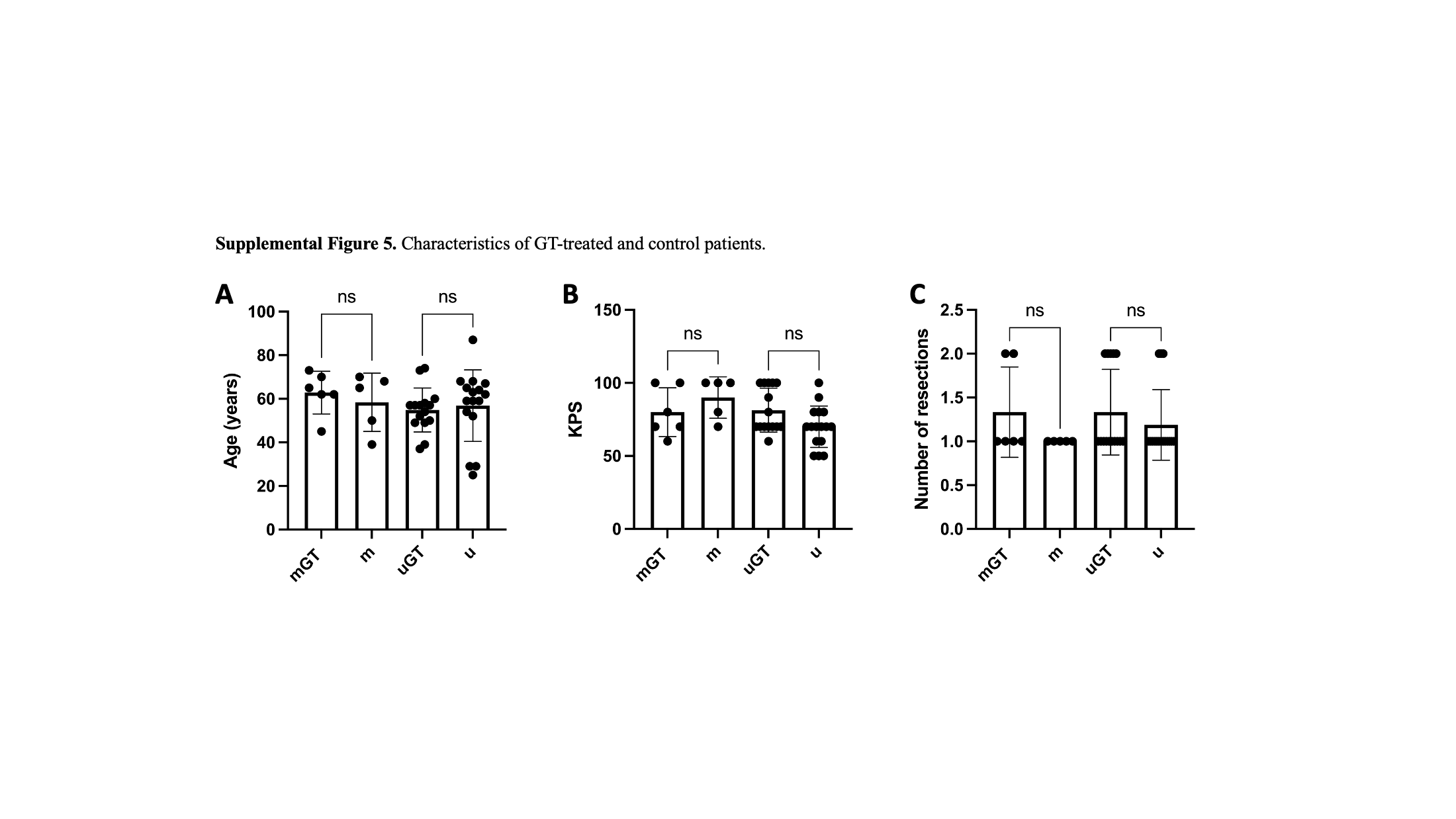

Supplement: vdab185_suppl_Supplementary_Material [file vdab185_suppl_supplementary_material.docx]
