# Supplementary material for: GammaTile® brachytherapy in the treatment of recurrent glioblastomas
Source: Neurooncol Adv. 2021 Dec 27;4(1):vdab185. doi: 10.1093/noajnl/vdab185 (PMC8788013; doi:10.1093/noajnl/vdab185)
Supplement: vdab185_suppl_Supplementary_Legends [file vdab185_suppl_supplementary_legends.docx]

**Supplemental Tables and Figures**

**Supplemental Table 1.** The oncologic treatment course for the GT-implanted patients and B) “control” cohort. The treatments were split into those received prior to and post-GT implant. The exact orders of treatment were not disclosed to minimize the risk of patient identification.

**Supplemental Table 2.** Post-operative patient characteristics and complications in patients with recurrent glioblastomas who underwent re-resection and GT placement.

**Supplemental Table 3**. The oncologic treatment course for the “control” cohort. The treatments were split into those received prior to and after resection only. The exact orders of treatment were not disclosed to minimize the risk of patient identification.

**Supplemental Table 4**. Demographic “control” patient cohort. A) MGMT methylated and B) MGMT unmethylated.

**Supplemental Figure 1.** GammaTile design. (**A**) Schematic GT, showing the dimension of the matrix and four titanium capsules containing^131^Cs seeds (gray). **(B)** Actual GT. GT is designed with a smooth and a studded surface. The studded surface faces the resection cavity to create an offset preventing seed-to-tissue contact and minimizing the risk of radiation-induced toxicity. (**C**) Hydrated GT becomes supple to conform to the resection bed's morphology but retains enough rigidity to maintain uniform or near uniform radiation-source spacing to avoid “hot” and “cold” spots in brachytherapy delivery.

**Supplemental Figure 2.** Time to repeat surgery for methylated (MGMTm) and unmethylated (MGMTu) glioblastomas.

**Supplemental Figure 3.** Correlation between GT parameters and local control (LC). **(A)** correlation between HR-CTV D_90_ and LC **(B)** correlation between HR-CTV V_50_ and LC **(C)** correlation between HR-CTV V_100_ and LC **(D)** correlation between HR-CTV V_150_ and LC. Linear regression R^2^ and associated p-values are shown.

**Supplemental Figure 4.** Tumor progression from residual tumor after GT placement. **(A)** Pre-operative MR post-gadolinium T1 images for patient 7. **(B)** post-resection MRI. Coronal slices through the anterior (top) and posterior margin (bottom) are shown. **(C)** HR-CTV of the GT (green). Residual tumor extends beyond the HR-CTV (red). **(D)** MRI post-gadolinium T1 taken 36 days post-resection/GT placement. MR perfusion imaging is shown below the axial MRI slice. Increased perfusion is seen in the anterior contrast enhancing region (yellow arrow). Coronal slices through the anterior (top) and posterior margin (bottom) are shown.

**Supplemental Figure 5.** Comparisons of age, KPS, and the number of previous resections between the GT-treated with or without MGMT methylation and control cohort with or without MGMT methylation. **(A)** comparison of age between the two cohorts. **(B)** comparison of KPS between the two cohorts. **(C)** comparison of the number of previous resections between the two cohorts. P-value derived based on student’s t-test.
